# Preventive Role and Survival Benefit of Beta Blockers in Pancreatic Cancer: A Systematic Review and Meta-Analysis of Observational Studies

**DOI:** 10.7759/cureus.104240

**Published:** 2026-02-25

**Authors:** Suprabhat Giri, Sidharth Harindranath, Prajna Anirvan, Manan Trivedi, Saroj K Sahu, Dibya L Praharaj, Bipadabhanjan Mallick, Preetam Nath, Sridhar Sundaram, Sarat Chandra Panigrahi, Manoj K Sahu

**Affiliations:** 1 Gastroenterology and Hepatology, Kalinga Institute of Medical Sciences, Bhubaneswar, IND; 2 Gastroenterology, Seth GS Medical College and KEM Hospital, Mumbai, IND; 3 Gastroenterology and Hepatology, Institute of Medical Sciences and SUM Hospital, Bhubaneswar, IND; 4 Surgery, K.B. Bhabha Hospital, Mumbai, IND; 5 Digestive Diseases and Clinical Nutrition, Tata Memorial Hospital, Mumbai, IND

**Keywords:** advanced pancreatic cancer, beta-adrenergic blocker, pancreatic-biliary cancer, survival analysis, systematic review and meta analysis

## Abstract

Beta-adrenergic receptor stimulation has been reported to positively influence the development and growth of many cancers in animal models. Studies have shown conflicting results regarding the benefit of beta-adrenergic receptor blockers in pancreatic cancer. Hence, we conducted a meta-analysis to investigate the relationship between beta-blocker usage and the prevention of pancreatic cancer and the prognosis after the diagnosis of pancreatic cancer. We searched electronic databases of Medline, Embase, and Scopus from January 2000 to August 2025 to identify studies reporting the relationship between beta-blockers and the development of new pancreatic cancer or survival in diagnosed cases of pancreatic cancer. Adjusted hazard ratios (aHR) were extracted for survival and pooled using a random-effects meta-analysis. One case-control and 13 cohort studies were identified, of which four analyzed the association between beta-blocker use and the incidence of pancreatic cancer, while the other 10 analyzed survival outcomes with the use of beta-blockers in patients with diagnosed pancreatic cancer. The pooled data showed that beta-blockers were significantly associated with a reduced incidence of pancreatic cancer (aHR = 0.77, 95% CI = 0.61 - 0.97, 3 studies). Similarly, continued use of beta-blockers after the diagnosis of pancreatic cancer was associated with improved survival (aHR 0.91, 95% CI: 0.87 - 0.94, 4 studies). However, the use of beta-blockers prior to diagnosis of pancreatic cancer did not improve survival (aHR 0.99, 95% CI: 0.94 - 1.05, 5 studies). The results of the current meta-analysis revealed that beta-blockers have a preventive and protective role against pancreatic cancer. Further research is required to validate the findings of this meta-analysis.

## Introduction and background

Pancreatic cancer remains a major global health challenge and is currently the seventh leading cause of cancer-related mortality worldwide [1]. Pancreatic ductal adenocarcinoma (PDAC), the predominant histologic subtype, is characterized by a dismal five-year survival rate of approximately 2-9%, largely attributable to aggressive tumor biology, a desmoplastic tumor microenvironment, and resistance to conventional systemic therapies [2-4]. These limitations have prompted increasing interest in identifying modifiable biological pathways and evaluating commonly prescribed medications as potential chemopreventive or adjunctive therapeutic agents.

β-adrenergic signaling has emerged as an important regulator of tumor initiation and progression. Chronic stress, smoking, and exposure to β-adrenergic agonists elevate circulating catecholamines, activating adrenergic receptors in tumor cells and the surrounding stromal microenvironment [5,6]. This activation stimulates intracellular pathways, including protein kinase A and mitogen-activated protein kinase signaling, leading to transcriptional activation of proliferative and pro-survival mediators such as nuclear factor-κB and cyclic adenosine monophosphate response element-binding protein [7,8]. Experimental evidence further indicates that β-adrenergic receptor 2 signaling promotes angiogenesis, immune modulation, and tumor-stromal interactions that facilitate PDAC progression [9,10]. In addition, sympathetic activation may contribute to pancreatic carcinogenesis through systemic inflammatory mechanisms involving alterations in the gut microbiome, impaired epithelial barrier integrity, and increased intestinal permeability, thereby promoting a pro-tumorigenic microenvironment [8,11-14]. β-blockers may counteract these effects by attenuating inflammatory signaling and restoring epithelial barrier function, providing a biologically plausible rationale for their potential antitumor role.

Despite compelling mechanistic data, clinical studies evaluating the role of β-blockers in pancreatic cancer have yielded inconsistent findings. While some observational analyses have suggested improved survival among β-blocker users, subgroup analyses comparing selective versus non-selective agents have not demonstrated a uniform benefit [15,16]. Consequently, the therapeutic and preventive implications of β-blocker exposure in PDAC remain uncertain. Therefore, the present systematic review and meta-analysis were undertaken to comprehensively evaluate the available evidence regarding the association between β-blocker use and (i) the incidence of pancreatic cancer and (ii) survival outcomes following PDAC diagnosis.

## Review

Methods

Information Sources and Search Strategy

We conducted a comprehensive search of all relevant studies using the databases of Medline, Embase, and Scopus from January 2000 to August 2025. The keywords used were ('beta adrenergic inhibitors' OR 'beta blockers' OR 'propranolol' OR 'metoprolol' OR 'atenolol' OR 'carvedilol') AND ('pancreatic cancer' OR 'pancreatic adenocarcinoma' OR 'pancreatic ductal adenocarcinoma'). To minimize the risk of missing eligible studies, the reference lists of all included articles were manually screened. The review was designed, conducted, and reported in accordance with the Preferred Reporting Items for Systematic Reviews and Meta-Analyses (PRISMA) statement [17].

Study Selection

All prospective and retrospective studies (both cohort and case-control) fulfilling the following PICO criteria were planned for inclusion. The meta-analysis is focused on two outcomes. The PICO criteria for the first outcome were: (i) patient: no prior history of pancreatic cancer, (ii) intervention: use of beta-blockers, (iii) controls: no treatment, and (iv) outcome: development of pancreatic cancer. The PICO criteria for the second outcome were: (i) patient: diagnosed cases of pancreatic cancer, (ii) intervention: use of beta-blockers, (iii) controls: no treatment, and (iv) outcome: survival. Studies were excluded if they were non-comparative in design, case series, review articles, or involved participants younger than 18 years. After applying predefined eligibility criteria, two reviewers independently screened the titles and abstracts of all retrieved records. Articles considered potentially eligible underwent full-text evaluation to confirm inclusion. Reference lists of eligible studies were also examined to identify additional relevant publications. Any discrepancies at any stage were resolved through discussion with a third reviewer.

Data Extraction and Study Quality Assessment

Data were independently collected by two investigators using a standardized extraction framework, with disagreements adjudicated by a third reviewer. Extracted variables included first author and publication year, country, study design, sample size, demographic characteristics, details of β-blocker exposure, outcome measures, and covariates included in adjusted analyses. The methodological quality of the included cohort studies was subsequently assessed using the Newcastle-Ottawa Scale (NOS) [18].

Statistical Analysis

Associations were summarized using adjusted hazard ratios (aHRs) with corresponding 95% confidence intervals (CIs). Pooled effect estimates were generated using a random-effects model and illustrated with forest plots. Where available, effect sizes derived from multivariable-adjusted models or propensity score-matched analyses were used. Between-study variability was quantified using the I² statistic, with values <25%, 25-75%, and >75% interpreted as low, moderate, and high heterogeneity, respectively [19]. Publication bias was evaluated using Egger’s regression test. Robustness of pooled estimates was examined through leave-one-out sensitivity analysis, sequentially omitting each study to assess its influence on the overall summary estimate. Statistical analyses were conducted using STATA version 17 (StataCorp, College Station, TX, USA).

Results

Study Characteristics and Quality Assessment

**Figure 1:**
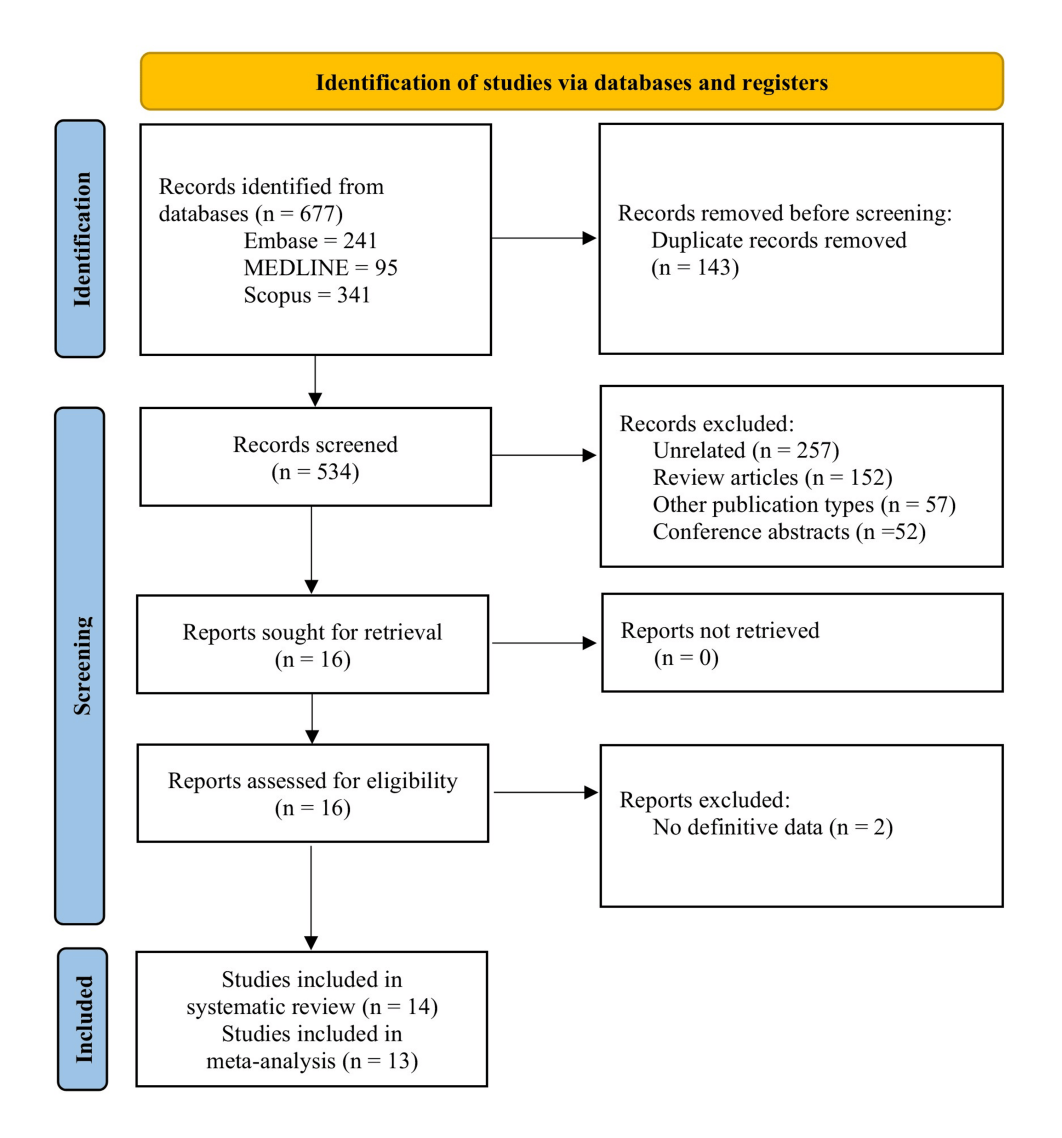
PRISMA flowchart for study identification, selection, and inclusion process Picture source: Dr. Suprabhat Giri

The search strategy identified 677 studies, 14 of which were included in the systematic review [20-33]. Figure 1 illustrates the PRISMA flow diagram outlining the study identification and selection process. The baseline characteristics of the included studies are summarized in Table 1. The number of patients in the included studies varied from 140 to 114669. Of the 14 studies, four reported on the preventive role of beta-blockers [20-23], among which one was a case-control study [22] and, hence, was not included in the meta-analysis. There was variation in the baseline patient population among these four studies. Wang et al. included only postmenopausal women [20], Kirkegard et al. (2019) included patients with chronic pancreatitis [21], while Cho et al. included patients with essential hypertension [23]. Ten studies reported on the survival effect of beta-blockers after diagnosis of pancreatic cancer [24-33]. Four studies included consecutive patients with a diagnosis of pancreatic cancer [24,26,31,33], two included only elderly patients [26,31], one included patients with advanced PDAC [28], one included middle-aged and elderly patients with pancreatic cancer [25], and two included only patients undergoing surgery [28,30]. Among the cohort studies, only one was prospective [20]. Table 2 shows the quality analysis for the included studies. Of these, eight were of good quality [22-24,26,27,29,31,33], and five were of medium quality [20,21,25,28,30,32].

**Table 1:** Baseline characteristics of the included studies BB: Beta-blockers; BMI: body mass index; CAD: coronary artery disease; COPD: chronic obstructive pulmonary disease; NSAID: non-steroidal anti-inflammatory drug; PDAC: pancreatic ductal adenocarcinoma; SBP: systolic blood pressure

Study	Country	Study design, duration	Patient characteristics	BB user	No BB user	BB type	Definition of BB use	Outcome	HR adjusted for
Prevention of pancreatic cancer									
Wang 2018 [20]	USA	Prospective cohort, 1993-1998	Postmenopausal women	11226	103443 (patients not on any antihypertensive drugs)	Mixed	Used for at least two weeks	Diagnosis of pancreatic cancer	Age at baseline, race/ethnicity, BMI, smoking status, alcohol consumption, self-reported type 2 diabetes at baseline, and self-reported hypertension at baseline
Kirkegård 2019 [21]	Denmark	Cohort, 1996-2012	Chronic pancreatitis, Males: 66.2%, 54 (IQR: 45-64) years	605	7706 (2660 on other and 5046 not on any antihypertensive drug)	Mixed	At least two filled prescriptions	Diagnosis of pancreatic cancer	Age, sex, socioeconomic status, year of chronic pancreatitis diagnosis, Gagne Comorbidity score, and other antihypertensive drugs
Saad 2020 [22]	UK	Case-control, 1995-2013	Individuals receiving medical care from a THIN (The Health Improvement Network) practitioner	4113 cases (males: 51.4%, 70.9±11.5 years) and 16072 matched controls (males: 51.5%, 71.1±11.4 years) (BB use in 1368 cases, and 4866 controls)		Mixed	Any prescription with BB prior to diagnosis of PDAC	First-time diagnosis of pancreatic cancer	Obesity, smoking, alcohol use, CAD, hypertension, diabetes, and CCB use
Cho 2021 [23]	Korea	Cohort, Jan 2005 to Dec 2017	Patients diagnosed with essential hypertension, Males: 60.9%, 55.2±9.2 years	13158	57391	Mixed	Prescription for at least 1 year	Diagnosis of pancreatic cancer	Age, sex, BMI, SBP, alcohol consumption frequency, income status, comorbidities (diabetes, heart failure, and COPD), and other antihypertensive drugs
Pancreatic cancer survival with BB exposure prior to diagnosis									
Shah 2011 [24]	UK	Retrospective cohort, 1997-2006	Individuals from the DIN-LINK primary care database	57	83	Mixed	Prescription in the 1-year period before diagnosis	All-cause mortality	Age, gender, year of diagnosis, smoking status, number of medications received in the year before diagnosis, area deprivation and national region
Springate 2015 [25]	UK	Retrospective cohort, Jan 1997 to Dec 2006	Patients aged 40–85 years with a first diagnosis of PDAC, Males: 55.3%	211	305	Mixed	At least 2 prescriptions in the 1-year period before diagnosis	Cancer-specific mortality	Age, gender, year of diagnosis, smoking status, number of medications received in the year before diagnosis, year of diagnosis
Udumyan 2017 [26]	Sweden	Retrospective cohort, 2006-2009	Patients with diagnosis of PDAC, Males: 48.7%, 67.9±9.7 years	522	1872	Mixed (Sub-group analysis)	At least one prescription within 90 days prior to diagnosis	Overall survival	Age, sex, attained education, healthcare/residence region, comorbidity score, TNM stage, tumor location in the pancreas, diagnosis year, b-blocker use, and medications
Yang 2021 [27]	USA	Retrospective cohort, 2007-2011	Patients aged 65 years and older with histologically confirmed PDAC, Males: 38.4%, 77.4±7.7 years	2564	4566	Mixed	Within 6 months prior to diagnosis of PDAC	Overall survival	Sex, age, marital status, race, income, Charlson comorbidity score, stage, & cancer-directed treatment
Huttner 2023 [28]	Germany	Retrospective cohort	Adult patients undergoing resection for pancreatic cancer, Males: 54.8%, 68 (IQR: 61-74) years	263	651	Mixed	Not defined	Overall survival	Age, sex, neoadjuvant therapy, ASA grade, AJCC grade, medications
Le Bozec 2023 [29]	Italy	Retrospective cohort, Nov 2015 to Jun 2022	Patients with advanced PDAC receiving intravenous anticancer treatment, Males: 54.9%, 65.73 ± 10.27 years	41	141	Mixed (Sub-group analysis)	Not defined	Overall and progression-free survival	age, gender, anticancer regimen, multimorbidity, polypharmacy, presence of cardiovascular comorbidity
Kirkegard 2023 [30]	Denmark	Retrospective cohort, 1997-2021	Patients aged 18 years or older with a record of resection for pancreatic cancer, Males: 54.8%, 68 (IQR: 61-74) years	433	2159	Mixed	Within 2 years prior to diagnosis	Overall survival	Sex, age, Nordic multimorbidity index score, hypertension, cardiovascular disease, alcohol intake, smoking, tumor stage
Pancreatic cancer survival with BB exposure after diagnosis									
Weberpals 2017 [31]	Netherlands	Retrospective cohort, Apr 1998 to Dec 2011	Patients with diagnosis of PDAC	113	141	Mixed	At least one BB any time after diagnosis within 12 months	Overall survival	Age, gender, TNM stage, socio-economic status at diagnosis, previous cancer, cardiovascular, cerebrovascular, diabetic, hypertensive, & pulmonary co-morbidities, year of diagnosis, concomitant medication use of NSAIDs, statins, antidiabetic, and antihypertensive
Beg 2018 [32]	USA	Retrospective cohort, 2006-2009	Individuals aged 66 or above with PDAC, Males: 42.5%, median 76 years	5209	8493	Mixed	At least 2 prescriptions filled within 12 months of PDAC diagnosis	Overall survival	Sex, race, stage, site, and Charlson comorbidity index
Stoer 2021 [33]	Norway	Retrospective cohort, Jan 2007 to Dec 2014	Patients aged 18–84 with diagnosis of PDAC, Males: 51.4%, median 67 years	411	2203	Mixed (Sub-group analysis)	Used BB both before and after PDAC diagnosis	Cancer-specific survival	Sex, age, comorbidity index, stage, statins, non-selective monoamine reuptake inhibitors, selective serotonin reuptake inhibitors and other Antidepressants,
Yang 2021 [27]	USA	Retrospective cohort, 2007-2011	Patients aged 65 years and older with histologically confirmed PDAC, Males: 38.4%, 77.4±7.7 years	1750	5335	Mixed	Used BB for at least 6 months before and after PDAC diagnosis	Overall survival	Sex, age, marital status, race, income, Charlson comorbidity score, stage, and cancer-directed treatment

**Table 2:** Newcastle-Ottawa quality assessment scale for cohort studies

Authors	Selection				Comparability	Outcome			Overall
	Representativeness of the exposed cohort	Selection of the non-exposed cohort	Ascertainment of exposure	Demonstration that outcome of interest was not present at the start of study	Comparability of cohorts on the basis of the design or analysis	Assessment of outcome	Was follow-up long enough for outcomes to occur	Adequacy of follow-up of cohorts	
Wang 2018 [20]		*		*	**	*	*	*	Fair
Kirkegård 2019 [21]		*		*	**	*	*	*	Fair
Saad 2020 [22]	*	*		*	*	*	*	*	Good
Cho 2021 [23]	*	*	*	*	**	*	*	*	Good
Shah 2011 [24]	*	*		*	**		*	*	Good
Springate 2015 [25]		*		*	**	*	*	*	Fair
Udumyan 2017 [26]	*	*		*	**		*	*	Good
Yang 2021 [27]		*	*	*	*		*	*	Good
Weberpals 2017 [31]	*	*		*	**		*	*	Good
Beg 2018 [32]		*		*	*		*	*	Fair
Stoer 2021 [33]	*	*	*	*	**	*	*	*	Good
Huttner 2023 [28]		*		*	*	*		*	Fair
Kirkegard 2023 [30]		*	*	*	*	*	*	*	Fair
Le Bozec 2023 [29]	*	*		*	*	*	*	*	Good

Effect of Beta-Blockers on the Prevention of Pancreatic Cancer

**Figure 2:**
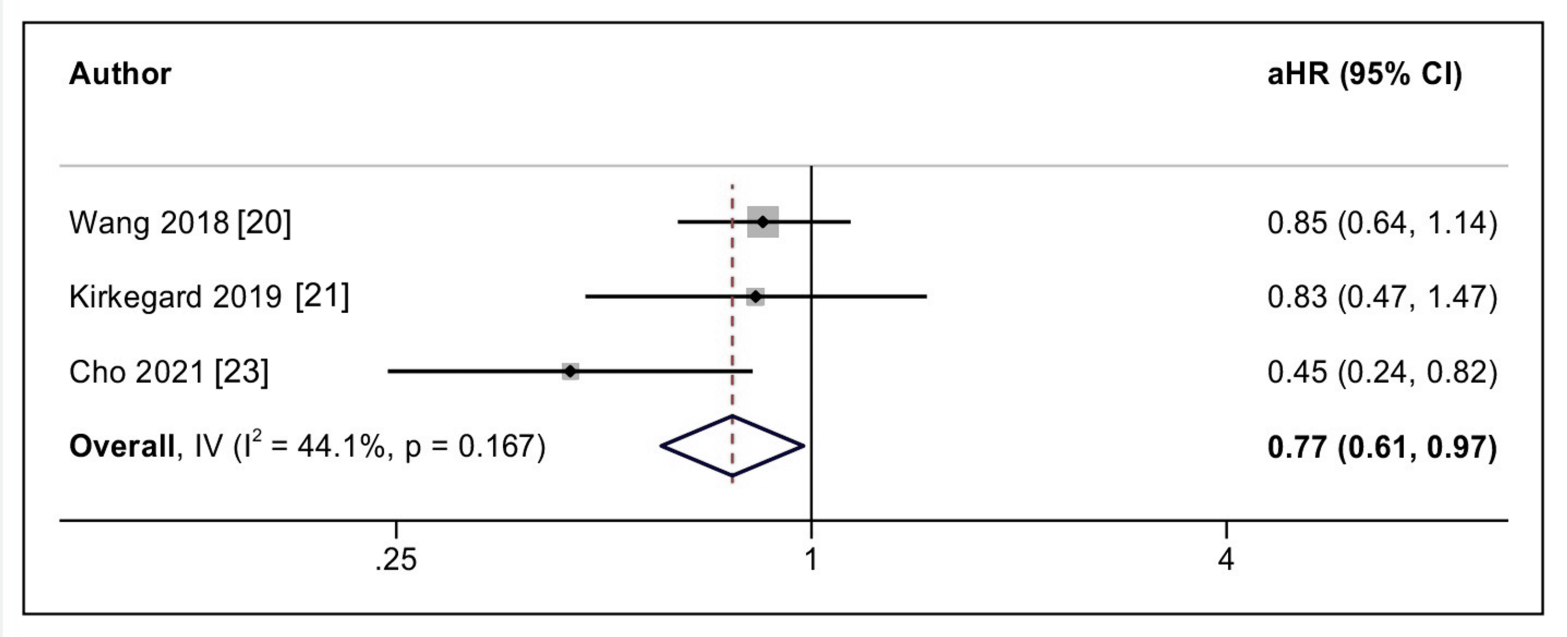
Forest plot for the prevention of the development of pancreatic cancer with the use of beta-blockers Picture source: Dr. Suprabhat Giri

The study by Wang et al. and Kirkegard et al. (2019) reported no benefit [15,16], while Saad et al. and Cho et al. reported the benefit of beta-blockers for the prevention of pancreatic cancer [17,18]. Saad et al. reported that only long-term beta-blocker use was associated with reduced pancreatic cancer risk. Three cohort studies were included in the final meta-analysis [15,16,18]. The included studies represented heterogeneous populations, including postmenopausal women, patients with chronic pancreatitis, and individuals with essential hypertension, each with differing baseline risks for pancreatic cancer development. The pooled results indicated that the use of beta-blockers was associated with a reduced incidence of pancreatic cancer (aHR = 0.77, 95% CI = 0.61 - 0.97; I2 = 44.1%) (3 studies, n = 193529), which was statistically significant (Figure 2).

Effect of Beta-Blockers on Survival in Patients With Pancreatic Cancer

Overall, 10 cohort studies reported on survival outcomes with the use of beta-blockers in patients with diagnosed pancreatic cancer. Of these 10 studies, six studies reported the association of survival with the use of beta-blockers prior to diagnosis of pancreatic cancer [19-21,23-25], three after diagnosis of pancreatic cancer [26-28], and one reported both [22].

**Figure 3:**
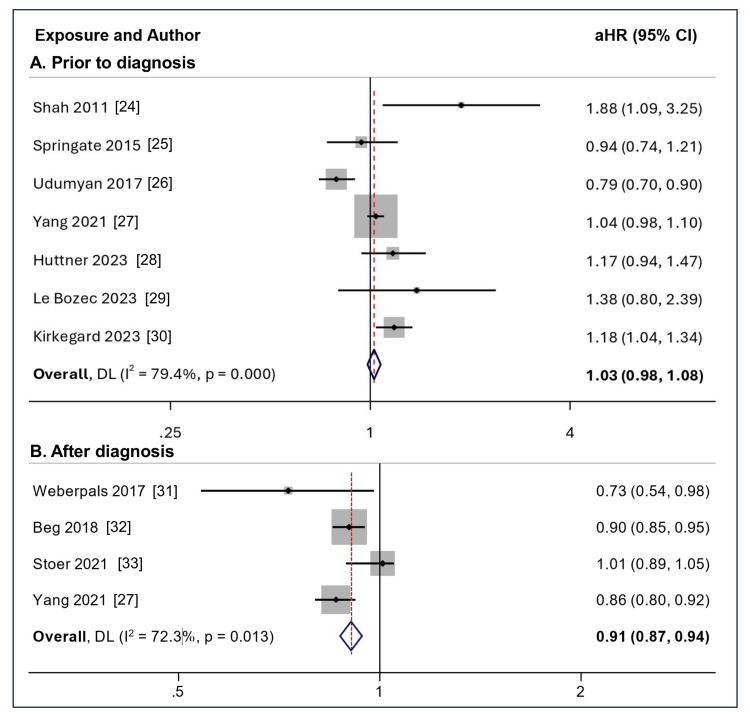
Forest plot for survival according to beta-blocker use (A) prior to the diagnosis and (B) after the diagnosis of pancreatic cancer Picture source: Dr. Suprabhat Giri

Among studies reporting the association of survival with the use of beta-blockers prior to diagnosis of pancreatic cancer, five studies reported no benefit [19,29,22-25], while only Udumyan et al. reported a survival benefit [21]. Kirkegard et al. (2023) analyzed the outcome of pancreatic cancer surgery in patients taking beta-blockers prior to the diagnosis of pancreatic cancer and reported increased mortality (aHR = 1.18, 95% CI = 1.04 - 1.34) [25], while Huttner et al. reported no increase in mortality [23]. The pooled data showed that the use of beta-blockers before the diagnosis of pancreatic cancer did not improve survival (aHR 1.03, 95% CI: 0.98 - 1.08; I2 = 79.4%) (7 studies, n = 11928) (Figure 3A).

Among studies reporting an association between survival and beta-blocker use after pancreatic cancer diagnosis, all except Stoer et al. [28] reported a survival benefit. The pooled data showed that continued use of beta-blockers after the diagnosis was associated with improved survival in patients with pancreatic cancer (aHR 0.91, 95% CI: 0.87 - 0.94; I2 = 72.3%) (4 studies, n = 23655), which was statistically significant (Figure 3B).

Publication Bias and Sensitivity Analysis

There was no evidence of publication bias on Egger's test for any of the outcomes (Table 3). Leave-one-out analysis did not show any significant change in the overall effect.

**Table 3:** Egger’s test for assessment of small study effect SE: Standard error

	β1	SE of β1	z	Prob > |z|
Effect of beta-blockers on the prevention of pancreatic cancer	-2.36	2.742	-0.86	0.3887
Effect of pre-diagnosis beta-blockers on the survival of patients with pancreatic cancer	1.87	1.063	1.76	0.0788
Effect of post-diagnosis beta-blockers on the survival of patients with pancreatic cancer	-1.70	1.515	-1.12	0.2626

Discussion

Stress-induced sympathetic nervous system activation (SNS) has recently been found to play an important role in the pathogenesis of various cancers, including pancreatic cancer. Stimulation of the sympathetic nervous system (SNS) releases catecholamines from the adrenal glands and the peripheral postganglionic sympathetic nerve fibers, which have been shown to increase angiogenesis, tumor cell growth, and survival as well as invasion, migration, and metastases in various cancers [5,15,16]. In recent years, there has been growing interest in using common cardiovascular medications, including beta-adrenergic receptor blockers, to improve the survival of patients with pancreatic cancer. This has led to multiple clinical and observational studies examining this outcome [34]. To the best of our knowledge, this is the first meta-analysis to comprehensively summarize the existing evidence on the effects of beta blockers on the prevention and improvement of survival in patients with pancreatic cancer.

In the present meta-analysis, pooled results indicate that the use of beta blockers was associated with a reduced incidence of pancreatic cancer (aHR = 0.77, 95% CI = 0.61 - 0.97). This is supported by data from various animal studies. Al-Wadei et al. showed the cancer-preventive effect of the non-selective beta blocker propranolol in hamsters prenatally exposed to ETOH/NNK (nicotinamide nitrosamine kinase) [35]. The study by Saad et al. demonstrated a lower risk of PDAC among long-term users, particularly those with more than 2 years of use, compared to never users. Even when former beta-blocker users were compared, the beneficial effect was observed only among long-term users [22]. Wang et al. studied the association between soluble receptor for advanced glycation end products (sRAGE) levels, antihypertensive medications, and the risk of developing pancreatic cancer [20]. sRAGE levels have been shown to be modulated by antihypertensive medications and inversely associated with the development of pancreatic cancer [36,37]. Wang et al. reported that beta-blocker users had a higher average sRAGE level than those on other anti-HT medications (1692 versus 1454 pg/mL, p > 0.05), which may contribute to a reduced risk [20]. However, this association should be interpreted with caution, as confounding by indication may have affected the results in many of the studies that looked at this question. Users of antihypertensive drugs may have higher baseline co-morbidities and hence a higher risk of pancreatic cancer. Conversely, individuals receiving antihypertensive therapy may demonstrate greater healthcare engagement, improved medication adherence, and more favorable health-related behaviors, which could independently influence outcomes.

In the present meta-analysis, pooled results showed that the use of beta-blockers prior to diagnosis of pancreatic cancer did not improve survival (aHR 1.03, 95% CI: 0.98-1.08). This result, consistent with Yang et al.'s study using the SEER database, showed that beta-blocker use in the six months prior to PDAC diagnosis did not confer a survival benefit [27]. The claim that beta-blockers improve survival in patients with PDAC is based mainly on preclinical studies. The few clinical studies that have assessed this parameter have shown conflicting results. In an analysis by Shah et al. using the UK Doctors’ Independent Network database, β-blocker exposure in the year preceding PDAC diagnosis was associated with reduced survival [24]. However, the study did not adjust for tumor stage or receipt of cancer-specific treatments. Consequently, the observed association may reflect residual confounding, particularly if patients receiving β-blockers had greater cardiovascular comorbidity and were less likely to undergo definitive oncologic therapy. Another study by Udumyan et al. demonstrated a survival benefit in patients with PDAC treated with beta-blockers, although this did not account for cancer-directed therapies [26]. Another reason for the difference in results might be the younger, healthier cohort of individuals in this study with early-stage disease (stage I-II). Any observed survival benefit attributable to β-blocker exposure may diminish or become clinically insignificant following the initiation of definitive cancer-directed therapies.

The present meta-analysis shows that continued use of beta-blockers after PDAC diagnosis was associated with a survival benefit (aHR 0.91, 95% CI: 0.87-0.94). In a large population-based cohort study of patients with PDAC, the use of beta-blockers was associated with a survival benefit after adjusting for age. This effect was most pronounced for patients with early localized disease. This indicates greater beta-blocker influence early in the clinical course and that beta-receptor inhibition is unlikely to affect progression once the tumor is established [26]. In a previous meta-analysis by Jiang et al., which included 12 studies comprising 120,549 patients, the use of antihypertensive medications had no negative effect on overall survival in patients with pancreatic cancer [37].

Most clinical studies examining the role of beta blockers in pancreatic cancer are based on epidemiological databases. These studies are inherently susceptible to immortal-time bias. Immortal time denotes a segment of follow-up during which the outcome event cannot occur by design of the study. If this interval is not appropriately accounted for in the analysis, it may artificially exaggerate a protective association of the exposure under investigation. Evaluations of post-diagnostic pharmacologic therapy in oncology are especially susceptible to this form of bias. Hence, this meta-analysis specifically aimed to account for this bias by including studies that looked at survival rates with exposure to beta-blockers both before and after the diagnosis of pancreatic cancer.

The strength of this meta-analysis lies in the inclusion of studies that examined the survival benefit of beta-blockers initiated before and after PDAC diagnosis. Secondly, we comprehensively reviewed studies assessing the preventive effect of beta-blockers on pancreatic cancer. There are also several limitations to the present meta-analysis. First, the majority of the studies included were observational, based on population registries, and were predominantly retrospective. Second, the included studies did not separately examine the effects of selective vs. non-selective beta-blockers. Thirdly, the analysis for the dose-response effect could not be carried out due to the paucity of literature. Fourth, other prognostic factors like lifestyle choices, smoking status, and CA19-9 levels were not included in the analyses. Fifth, differences in PDAC management across regions can vary widely and may affect patient survival. Lastly, the heterogeneity observed across included studies likely reflects differences in patient populations, disease stages, and indications for β-blocker therapy. Some cohorts comprised predominantly elderly patients with higher comorbidity burden and competing mortality risks, whereas others included patients with chronic pancreatitis, a biologically distinct population with increased baseline risk for pancreatic carcinogenesis. Indication bias may also have influenced outcomes, as β-blockers are commonly prescribed for conditions such as hypertension, ischemic heart disease, arrhythmias, or cirrhosis, each independently associated with prognosis. Furthermore, treatment duration and cumulative drug exposure were inconsistently reported, precluding assessment of dose-response relationships. Subgroup analyses or multivariable meta-regression were not feasible because individual patient-level data were unavailable and subgroup reporting was inconsistent across studies, representing an inherent limitation of this analysis and highlighting the need for future prospective studies with standardized reporting of β-blocker indication, exposure duration, and clinically relevant subgroups.

## Conclusions

In conclusion, the present meta-analysis suggests a protective effect of beta-blockers on the development of pancreatic cancer. Although no survival effect could be seen in patients using beta-blockers prior to the diagnosis of pancreatic cancer, the benefit was seen in patients who continued to take beta-blockers after the diagnosis. These findings are consistent with the biological premise that attenuation of β-adrenergic receptor-mediated signaling may modulate tumor progression in pancreatic ductal adenocarcinoma and raise the possibility that β-blockers could serve as adjunctive agents alongside established oncologic therapies. However, definitive conclusions require validation through well-designed prospective studies and adequately powered randomized controlled trials to determine their impact on survival outcomes in PDAC.
